# Eleventh international conference on progress in vaccination against cancer (PIVAC-11), 10–13 October 2011, Copenhagen, Denmark

**DOI:** 10.1007/s00262-012-1209-5

**Published:** 2012-02-05

**Authors:** Tania Køllgaard, Joost H. van den Berg, Marco Donia, Per thor Straten

**Affiliations:** Department of Hematology, 54P4, Center for Cancer Immune Therapy (CCIT), Copenhagen University Hospital, Herlev, Denmark

The eleventh scientific conference progress in vaccination against cancer (PIVAC-11) was held 10–13 October in Copenhagen, Denmark. Currently, the success of cancer immunotherapy as treatment modality is improving and newly approved drugs and immunotherapies are showing encouraging clinical results. To be effective, cancer vaccination needs to stimulate powerful immune responses against specific targets as well as overcome the barriers that cancer cells use to protect themselves. Especially, new drugs (i.e. Ipilimumab) have demonstrated that targeting suppressive elements leads to highly improved clinical outcome. PIVAC-11 was characterized by much optimism and interesting new data on how to improve cancer vaccination were presented. Talks were given on aspects of combination therapies, genetic vaccinations, combinations of chemotherapy and immunotherapy and tumor immune escape mechanisms.

The conference opened with a keynote evening, addressing the use of adoptive transfer of T cells in cancer patients. Patrick Hwu (Houston, TX, USA) presented the latest results of a phase II clinical trial based on adoptive cell therapy (ACT) with tumor infiltrating lymphocytes (TILs) at MD Anderson Cancer Center (MDACC). Thus far, 41 melanoma patients have been treated, 2/38 (5%) showed complete responses (CR) and 16/38 (42%) had a partial response (PR). The frequency of CD8^+^ T cells but not CD4^+^ T cells in TILs correlated with better clinical response (CR/PR). Intriguingly, the frequency of CD8^+^ T cells with surface expression of the inhibitory receptor “B and T Lymphocyte Attenuator” (BTLA) in TILs also correlated with better prognosis. Hwu pointed out that a major rate-limiting step in ACT seems to be the inefficient T-cell migration to tumors. His group has shown that melanoma specific T cells do not express CXCR2—the receptor for CXCL1 and CXCL8 expressed in melanomas, and the insertion of genes encoding this chemokine receptor into TILs may improve T-cell migration and result in better CR. Hwu presented data from murine model studies showing that gp 100-specific T cells transduced with CXCR2 (receptor for CXCL1 and IL-8) have enhanced accumulation in tumors, delay tumor growth and lead to improved survival. Furthermore, the combination of anti-PD-1 and BRAF inhibitors with ACT treatment is currently being investigated in mice. Interestingly, data showed that increased anti-tumor activity and enhancement of migration of T cells into tumors could be found.

Next, Ton Schumacher (Amsterdam, Holland) demonstrated—by analyses of more than 30 TIL products—that both the magnitude and frequency of tumor-specific T cells was very low in TILs. The group used a high throughput multimer-based method for T-cell detection (“combi-coding”) and analyzed the presence of T cells specific for a panel of 145 HLA-A2-restricted known CD8^+^ T-cell melanoma-associated epitopes [from both melanocyte differentiation (MD), cancer testis (CT) and overexpressed (OE) antigens]. It turned out that every patient had a unique combination of antigen-specific T cells. In addition, he demonstrated that specific T cells found in TIL products pre-therapy were also found in the periphery 1 month post-therapy. Indeed, TIL therapy seemed to broaden the melanoma specific T-cell repertoire as specific T cells found in TIL products and PBMC 1 month later were only rarely seen in pre-treatment PBMC. Interestingly, only the presence of T-cell reactivity against CT antigens showed a trend towards correlation with CRs (*p* = 0.12). Schumacher ended this section by mentioning that it will also be worthwhile to use this technology to examine the immunological consequences of future clinical approaches in melanoma such as the combination of Ipilimumab (anti-CTLA-4) and Vemurafenib (mutant BRAF inhibitor). In his second part, Schumacher elegantly demonstrated, by use of in vivo imaging, how sites of former infection or vaccination contained tissue-resident memory CD8^+^ T cells. These cells were dendritic cell shaped and seemed to function as skin patrols with crawling behaviour. Recognition of relevant antigen led to cessation of migration and the shape of the cells rounded up at the site of antigen presentation.

The conference now continued with other aspects of cell-based therapies and in addition to the use of TILs, T cells can also be manipulated before reinfusion to become tumor reactive. Marc Schmitz (Dresden, Germany) presented data from a clinical trial where chronic myeloid leukemia (CML) patients received a prophylactic infusion of in vitro generated tumor-reactive T cells after hematopoietic cell transplantation (HCT) was shown to lead to induction of peptide-specific cytotoxic CD8^+^ T cells in 7/14 CML patients. Leukemia-specific CD8^+^ T cells were generated by use of donor-derived dendritic cells loaded with leukemia antigens such as PR3-, WT1- and/or BCR-ABL-derived peptides. Schmitz demonstrated for the first time that prophylactic infusion of leukemia-specific T cells after HCT is feasible and safe and found that the probability of overall and molecular relapse-free survival was 93 and 49%, respectively. A new approach to adoptive immunotherapy was presented by Alexei Kirkin (Hørsholm, Denmark). By treating activated Th1 CD4^+^ T cells with a DNA-demethylating agent (5-aza-2′-deoxycytidine) expression of cancer/germline (CG) proteins was induced. These CD4^+^ T cells were then used instead of DCs for enrichment in vitro of autologous cytotoxic CD8^+^ T cells. A phase I trial is currently being carried out, 13 prostate cancer patients have received a single dose of cytotoxic lymphocytes with no signs of toxicity and 9 have had stabilization or decrease in PSA levels.

As a result of thymic selection, natural antitumor T cells exhibit weak affinity for their cognate peptide−MHC complexes and are therefore at a distinct disadvantage compared to their pathogen-specific counterparts. Andy Sewell (Cardiff, UK) discussed three novel strategies to overcome this obstacle. First, it was shown that altered peptide ligands could be used to influence the quality of a primed T-cell response through a process called TCR-optimised peptide skewing of the repertoire of T cells (TOPSORT). TOPSORT can selectively prime only the most effective TCR clonotypes to produce an overall more efficient response to tumor at the single cell level than priming with the natural antigen. Secondly, it was shown that T cells transduced with enhanced antitumor TCRs with affinities like the very best antiviral TCRs were more sensitive to antigen density and better at recognizing real tumor cells than T cells displaying equivalent natural affinity TCR. Thirdly, collaboration with Immunocore Ltd., has demonstrated high affinity soluble TCR fusion reagents consisting of an anti-CD3 scFV domain and monoclonal TCRs can redirect polyclonal T cells to lyse tumor cells and induce regression of established tumor. These ‘ImmTAC’ molecules are currently in phase I clinical trial.

The engineering of T cells with a recombinant chimeric antigen receptor (CAR) composed of antibody binding domains connected to domains that activate T cells is a novel and promising approach for the treatment of cancer that has already demonstrated clinical activity in chronic lymphocytic leukemia patients. Recent work by Hinrich Abken (Cologne, Germany) suggest CD20 to be a marker for melanoma stem cells that may account for <2% of all neoplastic cells. In mice cytotoxic T cells engineered with a CD20-targeting CAR eliminated this minor subset of cells as well as induced regression of whole melanoma lesions. The power of CAR T cells in an example of gastrointestinal carcinoma could be more improved by inducing IL-12 upon CAR engagement. He elegantly showed how antitumor-induced responses in mice mediated by macrophages are crucial for tumor control in this setting. This novel treatment by “T-bodies” with inducible IL-12 holds promise for effective treatment of malignant diseases.

Dendritic-based clinical vaccinations are currently being explored in clinical trials and new strategies were presented at the meeting. Gustav Gaudernack (Oslo, Norway) reported on their first phase I/II clinical trial targeting brain tumor stem cells by vaccination with monocyte-derived dendritic cells (DCs) transfected with autologous glioblastoma stem cell mRNA. He demonstrated that glioblastoma stem cells, a subpopulation of transformed cells that might be responsible for tumor propagation, resistance to conventional therapies and tumor recurrence, can be cultured in vitro in the majority (35/44) of patients and form neurospheres. In addition, in vitro neurosphere formation is an independent negative prognostic factor in glioblastoma. Treatment with transfected monocyte-derived DCs, given in combination with temozolomide and radiotherapy, generated tumor-specific immune responses in all treated patients, with no signs of severe toxicities. Gaudernack showed how, compared to disease-matched controls, patients had a 3.5 times longer progression free-survival. Another subset of DCs—the plasmacytoid (pDCs)—are also currently being used in clinical trials. To this end, Jolanda De Vries (Nijmegen, The Netherlands) described the first phase I clinical trial using FSME-IMMUN^®^ matured tumor-peptide-loaded pDCs isolated with the Clini-MACS and then injected into stage IV melanoma patients. Fifteen patients were treated and pDC vaccination appeared to be feasible and safe. Importantly, it was shown by use of scintigraphic imaging that upon injection pDCs migrated to distant lymph nodes.

The combination of vaccination with chemotherapy was reported by Leisha Emens (Baltimore, MD, USA). She investigated how to take advantage of simultaneous or sequential administration of cancer vaccines and conventional chemo- or targeted therapeutics by fostering immune responses for breast tumor rejection. By using the paired FVB/N—neu N mouse model of HER-2^+^ breast tumors (no immune tolerance/immune tolerance), they investigated mechanisms of synergy and antagonism between HER-2-targeted, granulocyte–macrophage colony-stimulating factor (GM–CSF)-secreting vaccines and sequentially-combined low dose of cyclophosphamide with doxorubicin or anti HER-2 monoclonal antibodies. This revealed a synergistic activity between the vaccine and chemotherapies in both cases. Indeed, she showed how these drug combinations boosted vaccine-induced immunity. She subsequently discussed the results of two clinical trials demonstrating the safety and bioactivity of these combination vaccine approaches in both metastatic HER-2-negative (cyclophosphamide and doxorubicin) and HER-2-positive (cyclophosphamide and the anti-HER2 antibody trastuzumab) breast cancer. Another example of combination strategies was presented by Esteban Celis (Tampa, FL, USA) demonstrating that cancer vaccines via the co-administration of peptide vaccines with Poly-IC and anti-CD40 mAb (so-called TriVax) and a newer approach solely using peptide and Poly-IC (BiVax) can induce powerful T-cell responses in mice that lead to significant anti-tumor effects. However, the success of the BiVax strategy depends on the physical characteristics of the peptide; he believes that only those peptides that are able to form a complex with the Poly-IC (via ionic or hydrophobic interactions) may exhibit this high immunogenicity. This knowledge can be used to modify non-immunogenic peptides into high immunogenic variants. Furthermore, he presented interesting data showing that the success of peptide vaccination in mice is inhibited by IFN-γ. Tumors exposed to IFN-γ evade CTLs by expressing non-cognate major histocompatibility complex class I molecules, which limit T-cell activation and effector function. Indeed, adjuvants can be an essential part of an effective vaccine and Else Marie Agger (Copenhagen, Denmark) presented a new generation adjuvant system that involves a delivery vesicle to promote uptake and presentation of a vaccine antigen. The cationic liposomal adjuvant CAF01 has been shown to induce both humoral and cellular responses and is currently used in several phase I clinical trials. Incorporation of the TLR3 ligand polyinosinic:polycytidylic acid (pI:C) in the CAF01 lipids was shown to induce CD8^+^ T cells to high levels, and these T cells were demonstrated to be cytolytic.

The use of a DNA vaccine strategy encoding CTL epitopes within the CDR of an antibody (ImmunoBody™) was presented by Lindy Durrant (Nottingham, UK). The ImmunoBody DNA vector platform stimulates antitumor activity using mAbs genetically engineered to present CTL epitopes and effectively targeting DCs by cross presentation via the CD64 (FcgR1) receptor on DC. Lindy elegantly showed that vaccination with Immunobody™ induces high frequency and moderate avidity tumor-specific CD8^+^ and CD4^+^ responses in mice with high cancer killing capacity. Interestingly, these responses could not be increased by the APCs activator Homspera^®^ but this compound could enhance anti-tumour responses. Recently, a phase I/II trial in stage III/IV melanoma involving 4 UK centers was started.

The immunological monitoring of responses after clinical vaccination and correlation to outcome is essential for our future understanding of the interplay between vaccination, anti-tumor immunity and immune suppressive mechanisms. As demonstrated by Cécile Gouttefangeas (Tübingen, Germany) multipeptide vaccination including both CTL and Th epitopes (phase I/II trial) induced peripheral T cells against the majority of the peptides used for vaccination in 37 prostate cancer patients. Prostate specific antigen (PSA) doubling times were measured as a marker for tumor response, and interim results showed that patients receiving TLR7/8 adjuvants had a better clinical course. Furthermore, she presented a status report on the “cancer immunotherapy immune-guiding program” (CIP) which currently has 43 participating laboratories. The mission of CIP is to harmonize and increase the quality of in vitro monitoring assays used in different laboratories. One instrumental tool to reach this objective is to organize regular international proficiency panels for T-cell assays. The upcoming proficiency panels will fall in three categories. (1) Annual large scale service panels for performance feedback, (2) small scale development panels for specific questions/tests and (3) small scale exploratory panels for testing new assays. Graham Pawelec (Tübingen, Germany) presented the immunological monitoring data of a pilot RNA vaccination trial in melanoma. In this study, stage III and IV melanoma patients were vaccinated with an RNA vaccine encoding the antigens NY-ESO1, Survivin, MAGE-A3 and Melan-A. The immune monitoring showed a significant association between survival of unresectable stage IV patients and the presence of T cells responding to NY-ESO-1 or Melan-A peptides. On the other hand, no association between survival and the presence of T cells responding to Survivin or MAGE-A3 could be detected. The survival rates were even higher in patients that responded to multiple peptides and in patients that showed an unopposed pro-inflammatory response. Interestingly, only CD8^+^ T-cell reactivity to Melan-A correlated with survival, whereas both CD4^+^ and CD8^+^ reactivity to NY-ESO-1 correlated equally well with survival. In the last part of the presentation, Pawelec discussed the possible correlation between CMV status and responsiveness to immune therapy. The incidence of CMV seropositivity is especially high in the elderly in whom CMV control requires a huge commitment of immune resources. In the presented clinical trials, a correlation between NY-ESO1 response in the prospective long term survival group (>18 months) and a negative CMV status was observed, indeed, suggesting that the measurement of CMV seropositivity should be included in future immunomonitoring.

Immunosenescence in elderly people has been shown to be characterized in part by decreased numbers of naïve T cells as well as decreased diversity and function of memory T cells. As discussed by Claudia Gravekamp (New York, NY, USA) in the Joseph Lustgarten Memorial Lecture a novel recall approach using Listeria to selectively infect and kill tumor cells may overcome the compromised immunity. She started out by remembering Joseph Lustgarten who sadly passed away recently, and acknowledged his scientific contributions to cancer immunology and ageing. This led to presentation of her own work on a Listeria-based vaccine. Listeria infects both monocytic and granulocytic myeloid derived suppressor cells (MDSC) and as MDSC accumulate in tumors they will bring Listeria, which is then capable of infecting and killing tumor cells directly. Importantly, MDSC in the tumor microenvironment are highly immune-suppressive and will protect Listeria from clearance by immune cells. Indeed, healthy tissues are not immune suppressed and Listeria will be effectively cleared. Furthermore, the Listeria used can be modified to express T-cell epitopes of recall antigens, such as tetanus toxoid (TT). As shown in mice studies, when using Listeria-TT in mice pre-vaccinated with TT, tumor cells were also killed by TT antigen-specific memory T cells. This recall approach to activate T cells already present from childhood vaccination will further overcome the needs for naïve T cells in the response to vaccination of older patients.

Indeed, a great number of tumor specific peptides have been identified and post-translational modifications and altered glycosylation in tumor cells shown to lead to generation of new tumor epitopes. Victor Engelhard (Charlottesville, VA, USA) discussed how phosphorylation of peptides creates new tumor antigens that can provide novel targets for immunotherapy and cancer vaccination. These so-called phosphopeptides are formed post-translationally by the degradation of properly folded proteins within the cell and several phosphopeptides are upregulated in cancer. 3D analysis of MHC interactions showed that phosphopeptides can be recognized by T cells and can alter MHC binding of the original peptide, thereby creating new antigens. As presented by Ana Maria Vazquiz (Havana, Cuba), pre-clinical and clinical trials with Racotumomab; an anti-idiotype mAb directed against NeuGc-containing gangliosides expressed in tumors, showed that Racotumomab reduces lung metastases incidence in murine lung and breast carcinomas by increasing tumor cell apoptosis, increasing the number of infiltrating CD4^+^ and CD8^+^ lymphocytes and reducing the number of blood vessels. Clinical data showed that Racotumomab is well tolerated in patients. In a randomized, double blind, placebo-controlled phase II/III efficacy clinical trial in advanced NSCLC the intent to treat analysis (ITT) showed a statistically significant benefit in overall survival for the Racotumomab arm. At this point a phase III multinational clinical trial is ongoing.

Even though vaccination may lead to induction of successful anti-tumor immune responses, tumor escape mechanisms have been shown to protect malignant cells from immune recognition and strategies to overcome these major obstacles to successful clinical vaccination were presented and discussed. Barbara Seliger (Halle, Germany) addressed the down-regulation of HLA class I molecules on tumors. She demonstrated that impaired JAK2 expression leads to reduced IFN-γ based inducibility of the expression of HLA class I antigen processing machinery (APM) in melanoma cell lines. Furthermore, she showed that the transcription factor E2F1 was a central regulator of the HLA class I APM components and, consistent with this, deregulation of E2F1 has been observed in many cancers. As suggested by Federico Garrido (Granada, Spain), immunotherapy changes the tumor microenvironment which leads to HLA class I negative metastases. Tumors with dysregulation of HLA class I can be classified as either (1) soft lesions that up regulate class I in response to therapy or (2) hard lesions having irreversible low expression of HLA class I and being resistant to therapy. The nature of the preexisting HLA-class I lesions may play an important role for determining the final outcome of cancer immunotherapy. Garrido showed that IFN or vaccination lead to immune selection of a subgroup of metastases with irreversible low HLA class I expression (hard lesions).

MDSC are known to accumulate in tumors and have a direct effect on the suppression of tumor-reactive T-cells, the polarization of macrophages towards a tumor-promoting M2-like phenotype, and the trafficking of T cells. Suzanne Ostrand-Rosenberg (Baltimore, MD, USA) showed how the MDSC levels in mice could be regulated by T cells expressing Fas Ligand (FasL). Via Fas–FasL interactions T cells were capable of killing Fas^+^ MDSC in vivo, thus, suggesting a “retaliatory relationship” between T cells and MDSC. Thus, MDSC suppress T-cell activation; however, when T cells are activated they can trigger MDSC apoptosis. Based on gene expression profiling Thomas Gajewski (Chicago, IL, USA) described two categories of metastases from advanced melanoma patients. One subgroup had an inflamed phenotype expressing chemokines and T-cell markers, as well as other immunoregulatory factors. Indeed, this subgroup showed the highest expression of negative regulators such as PD-L1, Indoleamine 2,3-dioxygenase (IDO) and FoxP3, also these metastases were rich in CD8^+^ T cells. In contrast, the non-inflamed metastases lacked chemokines for T-cell migration and were T cell-poor. Furthermore, he showed that anergic T cells in tumor settings have surface expression of LAG3 and CRTAM. Gajewski asked: which came first? Do T cells coming to tumors induce suppressiveness (e.g. upregulation of PD-L1, IDO) or are tumors suppressive prior to T-cell migration? His group’s data suggest that upregulation of IDO and PD-L1, and recruitment of Tregs, in the tumor microenvironment are dependent on CD8^+^ T cells and therefore immune-intrinsic. As shown by Vincenzo Bronte (Verona, Italy) expression of the CCL2-receptor; CCR2, by T cells was essential for their intra-tumoral localization. Reactive nitrogen species (RNS) in the tumor lead to nitration of the chemokine CCL2 resulting in decreased recruitment and infiltration of T cells into murine and human tumors. The use of a drug (AT38) inhibiting the intratumoral generation of RNS facilitated the invasion of tumor-specific T cells into tumors after adoptive cell transfer (ACT) with mTERT-reactive CTL in mice. Better survival of these mice indeed suggested that targeting of RNS production in tumors may help improve the efficacy of cancer immunotherapy.

Immunoregulatory cells named supporter T cells (Tsup) were presented by Mads Hald Andersen (Herlev, Denmark) as T cells specific for IDO. They can be detected in cancer patients and healthy volunteers and have been observed in both CD4^+^ and CD8^+^ T-cell populations. IDO-specific CD8^+^ T cells are able to kill IDO-expressing tumor cells and DCs. Intriguingly, IDO-specific CD8^+^ T cells can boost T-cell immunity against other antigens in vitro as well as reduce the level of Tregs in cultures. Thus, they seem to overcome or delay the immune suppressive actions of the IDO-protein when expressed by maturing APC and as a consequence the IDO-specific CD8^+^ T cells have been thus named. In addition to IDO, T-cell reactivity against other immunoregulatory molecules, such as Indoleamine 2,3-dioxygenase-2 (IDO2), heme oxygenase-1 (HO-1) and NF-kB-inhibitor has also been observed, indicating that self-reactive antigen-specific T cells recognizing immune regulatory targets have an immune regulatory role. Sjoerd van der Burg (Leiden, Holland) described how CD8^+^ and CD4^+^ T cells found in TILs and tumor draining lymph nodes (TDLN) from cervical carcinoma patients were polyclonal and reactive against a broad HPV antigen repertoire. He demonstrated that soluble factors secreted by cervical carcinomas induced IL-10-producing M2 macrophages and, interestingly, IFN-γ-producing CD4^+^ T cells could switch M2 macrophages to M1 macrophages. This surely suggests that boosting HPV-specific CD4^+^ Th1 immunity is relevant in cancer therapy; however, it should be considered that HPV-specific CD4^+^ Tregs may be induced as well which could lead to clinical failure.

Effective cancer vaccination appears to require T-cell immunity, however, there is still a lack of correlation between induction of tumor responses in patients with their clinical outcome. At the PIVAC-11 meeting new strategies and approaches were presented addressing the issues of identifying new tumor antigens, promoting powerful tumor specific T-cell responses and immune monitoring. It has become clear that immune escape mechanisms and immune suppression are major obstacles to successful cancer treatment and the combination of cancer vaccination with targeting of the immune suppressive elements holds great promise to future cancer treatment. To this end, it is important to learn more about the plasticity of immune regulatory cells and how they are induced in different tumor microenvironments.

PIVAC-12, the next scientific meeting in this series, will take place in Nottingham, UK, from 11 to 13 September, 2012.

